# Investigating the Impact of Body Composition Analysis on Quality of Life and Anxiety–Depression in Adult Males with Chronic Obstructive Pulmonary Disease

**DOI:** 10.3390/healthcare13121442

**Published:** 2025-06-16

**Authors:** Ahmet Kurtoğlu, Özgür Eken, Rukiye Çiftçi, İpek Balıkçı Çiçek, Dilber Durmaz, Mine Argalı Deniz, Monira I. Aldhahi

**Affiliations:** 1 Department of Coaching Education, Sport Science Faculty, Bandırma Onyedi Eylul University, Balıkesir 10250, Turkey; akurtoglu@bandirma.edu.tr; 2 Department of Physical Education and Sport Teaching, Sport Science Faculty, Inonu University, Malatya 44280, Turkey; ozgureken86@gmail.com; 3 Department of Anatomy, Medical Faculty, Gaziantep Islam Science and Technology University, Gaziantep 27010, Turkey; rukiyekelesciftci@hotmail.com; 4 Department of Biostatistics and Medical Informatics, Faculty of Medicine, Inonu University, Malatya 44280, Turkey; ipek.balikci@inonu.edu.tr; 5 Department of Thoracic Diseases, Balikesir, Medical Faculty, Bandirma Onyedi Eylul University, Bandırma 10250, Turkey; dilberdurmaz@bandirma.edu.tr; 6 Department of Physical Therapy and Rehabilitation, Suleyman Demirel University Research and Application Hospital, Isparta 32100, Turkey; minedeniz@sdu.edu.tr; 7 Department of Rehabilitation Sciences, College of Health and Rehabilitation Sciences, Princess Nourah bint Abdulrahman University, P.O. Box 84428, Riyadh 11671, Saudi Arabia

**Keywords:** COPD, dyspnea, daily living activity, fatigue, chronic respiratory disease, anxiety, depression

## Abstract

Background/Objectives: Chronic obstructive pulmonary disease (COPD) is a progressive respiratory disorder characterized by systemic manifestations, including altered body composition, reduced quality of life, and psychological distress. Despite its significance, the relationship between body composition parameters and symptoms of fatigue, anxiety, and depression in patients with COPD remains underexplored. This study aimed to examine the association between detailed body composition metrics and quality of life, fatigue, and anxiety and depression symptoms in male patients with COPD compared to healthy controls. Methods: This cross-sectional study included 49 men with COPD and 51 age-matched healthy controls aged 50–80 years. Body composition was assessed using bioelectrical impedance analysis (BIA). Pulmonary function, dyspnea, activities of daily living, and psychological status were evaluated using spirometry, the Medical Research Council Dyspnea Scale, the London Chest Activity of Daily Living Scale (LCADL), and the Hospital Anxiety and Depression Scale (HADS), respectively. Results: Compared to the controls, patients with COPD exhibited significantly lower forced expiratory volume in one second (FEV_1_: 1.1 vs. 2.16 L; *p* < 0.001), lower fat mass (15.0 vs. 24.3 kg; *p* < 0.001), and higher muscle mass (53.8 vs. 42.0 kg; *p* < 0.001). They also reported significantly greater fatigue (Borg scale: 4 vs. 0; *p* < 0.001), higher anxiety (8 vs. 5; *p* = 0.006), and depression scores (11 vs. 5; *p* < 0.001), along with more pronounced limitations in their daily activities. Conclusions: COPD is associated with profound impairments in body composition, physical function, and mental health. Detailed body composition analysis using BIA provides valuable clinical insights and may aid in tailoring individualized interventions to improve quality of life and psychological outcomes in COPD management.

## 1. Introduction

Chronic obstructive pulmonary disease (COPD) is a common and progressively debilitating respiratory condition, primarily characterized by persistent airflow limitation and chronic airway inflammation [1]. According to the Global Burden of Disease study, COPD is currently the third leading cause of death worldwide, posing significant individual and public health challenges [2]. Among its most prominent risk factors, habitual tobacco smoking remains the primary contributor to disease development and progression [3]. Epidemiological data consistently show that COPD is more frequently diagnosed in men than in women [4]. Importantly, patients with COPD often experience significant systemic manifestations, irrespective of the severity of airflow obstruction. Notably, unintentional weight loss and reductions in lean muscle mass are prevalent and clinically consequential issues that undermine physical performance and functional capacity [5]. Systemic inflammation in COPD has been implicated in the development of cachexia, anorexia, skeletal muscle dysfunction, and worsening dyspnea [6]. These pathophysiological changes collectively lead to a deterioration in overall health status and an elevated risk of disease exacerbations and are strongly associated with increased mortality rates [7,8].

Skeletal muscle weakness affects approximately 32% of individuals with COPD [9], and its prevalence tends to increase with disease severity, regardless of pulmonary function [10]. Dyspnea on exertion, muscular fatigue, and exercise intolerance contribute to the progressive deterioration of functional capacity. Upper limb muscle weakness is frequently observed in patients with COPD and is particularly detrimental to activities requiring arm elevation and endurance [11]. Consequently, everyday tasks such as walking, stair climbing, and upper-body-related activities of daily living (ADLs)—including dressing, bathing, grocery shopping, and routine household chores—are significantly impaired [12].

There is a growing emphasis on evaluating body composition in patients with COPD, with particular attention to fat mass, owing to its well-established impact on health outcomes. Excess adiposity is associated with a worsened prognosis and reduced treatment efficacy. While earlier assessments relied on simple anthropometric indices, such as the weight-to-height ratio, recent advancements have favored more precise techniques [13]. The Tanita body fat analyzer, which operates on the principle of bioelectrical impedance analysis (BIA), offers a rapid and noninvasive method for detailed body composition assessment [14]. This device enables clinicians to obtain comprehensive estimates of fat mass and other body compartments, thereby supporting more individualized management approaches [15].

In practice, BIA, or body composition measurement, should be inexpensive, non-invasive, operator-friendly, and provide highly reproducible and accurate results [16]. The assessment methods that show the most accurate and reliable results are computed tomography (CT), hydrostatic weighing, and dual-energy X-ray absorptiometry (DEXA) [17]. Therefore, these methods are considered reference standards. However, the problem with this technology is that it is prohibitive in most practical cases owing to the high costs and the need for laboratory space, operator training, and experience. Furthermore, these methods do not eliminate the possibility of measurement errors [18].

COPD not only affects the lungs and airways but also causes nutritional abnormalities, such as weight loss, increased resting energy expenditure, and/or abnormal body composition. These conditions produce fatigue in patients with COPD [19]. Fatigue is another prevalent symptom in patients with COPD; however, it remains one of the most under-recognized and undertreated aspects of the condition. It is often misinterpreted as dyspnea, which can lead to suboptimal therapeutic strategies that only marginally alleviate symptom burden [20]. Persistent fatigue may contribute to the development of mood disorders, particularly depression and anxiety, which are common comorbidities of COPD [21].

These psychological disorders are associated with poorer clinical outcomes, including reduced survival, prolonged hospitalization, and increased mortality risk [22]. Epidemiological studies indicate that the prevalence of clinical depression in stable COPD ranges from 10% to 42%, while anxiety affects 10% to 19% of patients [23]. According to the stress-coping framework proposed by Lazarus and Folkman, maladaptive coping mechanisms in patients with COPD significantly hinder psychological adaptation and overall quality of life [24].

Despite the increasing use of BIA in COPD research, comprehensive studies examining the relationship between BIA-derived body composition parameters and physical performance, daily functioning, and psychological well-being in COPD populations are lacking. This gap is particularly pronounced in male patients, who are often underrepresented in studies focusing on systemic outcomes of COPD. Therefore, this study aimed to evaluate the relationship between detailed body composition metrics and quality of life, fatigue, and symptoms of anxiety and depression in male patients with COPD using a multidimensional assessment approach and comparison with healthy controls. We hypothesized that male patients with COPD would exhibit significantly altered body composition, characterized by reduced fat mass and increased fluid retention and muscle mass, as well as poorer outcomes in quality of life, fatigue, and psychological well-being (anxiety and depression) compared to age-matched healthy controls.

## 2. Materials and Methods

### 2.1. Study Design and Participants

This study included 49 male patients diagnosed with chronic obstructive pulmonary disease (COPD group) and 51 age-matched healthy male participants (control group, CG), all aged 50–80 years. A priori power analysis was conducted using G*Power software (version 3.1.9.7, University of Düsseldorf, Germany) to determine the required sample sizes. Based on an alpha level of 0.05, an effect size of 0.50, and a desired statistical power of 0.80, the minimum number of participants needed was calculated to be 90, ensuring an actual power of 80.5%. Accordingly, 100 eligible participants were recruited for the study. COPD diagnosis was established according to the Global Initiative for Chronic Obstructive Lung Disease (GOLD) criteria, with participants exhibiting a post-bronchodilator FEV1/FVC ratio of <0.70. The inclusion criteria for the COPD group were age between 50 and 80 years, literacy, ability to ambulate independently, no cognitive impairments, and no history of thoracic surgery. For the control group, the inclusion criteria were being within the same age range, being literate, and being free from any diagnosed pulmonary, cardiac, or orthopedic conditions. The exclusion criteria for both groups encompassed chronic asthma, significant musculoskeletal disorders, acute infections, metabolic or cardiovascular diseases, other chronic respiratory illnesses, and the use of beta-blocker medications.

This study was conducted in accordance with the principles of the Declaration of Helsinki. Ethical approval was obtained from the Ethics Committee of the Institute of Health Sciences at the Bandırma Onyedi Eylu University (approval number: 2022/15).

### 2.2. Study Procedure

#### 2.2.1. Pulmonary Function Tests

Pulmonary function tests (PFTs), which provide objective measurements of respiratory function, were performed using a portable spirometer (Spirolab; SDI Diagnostics, South Easton, MA, USA). All assessments were conducted at room temperature (approximately 25 °C) under standardized conditions. The key spirometric parameters recorded included peak expiratory flow (PEF), forced expiratory flow at 25% to 75% of vital capacity (FEF25–75), and forced expiratory volume in one second (FEV_1_). Maximum voluntary ventilation (MVV) was assessed using an alternative spirometric protocol. All procedures adhered to established international acceptability and reproducibility criteria, ensuring the reliability and consistency of the test results across participants [25].

#### 2.2.2. Medical Research Council (MRC) Dyspnea Scale

Perceived dyspnea was evaluated using the Medical Research Council (MRC) dyspnea scale, which is a validated instrument for assessing the severity of breathlessness in individuals with respiratory conditions [26]. Participants provided self-reported ratings of their breathlessness during daily activities and were subsequently categorized into MRC grades 0–5, reflecting increasing levels of functional impairment. A score of 0 indicates dyspnea only during strenuous physical exertion, whereas a score of 5 indicates severe breathlessness that occurs during basic tasks, such as dressing or leaving the house [25]. This scale enables stratification of symptom severity and supports the evaluation of disease burden in patients with COPD.

#### 2.2.3. London Chest Activity of Daily Living Scale (LCADL)

The London Chest Activity of Daily Living Scale (LCADL) is a simple and standardized questionnaire developed by Garrod et al. to assess dyspnea caused by daily living activities in patients with COPD. This 15-item questionnaire comprises four components: personal care (4 items), household chores (6 items), physical activity (2 items), and leisure time (3 items). Each item was scored on a scale of 0–5: 0 (I do not do this activity because I have never had to do it or it is irrelevant), 1 (I do not feel breathless at all when doing this activity), 2 (I feel moderately breathless when doing this activity), 3 (I feel very breathless when doing this activity), 4 (I cannot do this activity because of my shortness of breath and there is no one who can do the activity for me), and 5 (I can no longer do this activity, and I need someone to do it for me or help me because of my shortness of breath). Higher scores indicate greater limitations in the daily activities. The scale can be evaluated in terms of the total, component, and individual item scores. The total score can reach a maximum of 75 points [5].

#### 2.2.4. Hospital Anxiety and Depression Scale (HAD)

Sociodemographic characteristics, including age, marital status, educational level, occupation, psychiatric history, family history of psychiatric disorders, and alcohol or substance use, were collected using a standardized sociodemographic form. Although the patients were initially screened for alcohol or substance dependence, none met the diagnostic criteria for dependence. Nevertheless, participants were asked about their use of alcohol, tobacco, and other substances. Psychological distress was assessed using the Hospital Anxiety and Depression Scale (HAD), a self-administered screening tool widely used in clinical settings to evaluate symptoms of anxiety and depression. The scale comprises two subscales—anxiety and depression—each containing seven items, with a cutoff score of ≥8 indicating clinically relevant symptoms. Each item was answered by the patient on a four-point (0–3) response category, so that possible scores ranged from 0 to 21 for anxiety and 0 to 21 for depression. Analysis of scores on the two subscales in a further sample in the same clinical setting provided information that a score of 0–7 for both subscales could be considered within the normal range, a score of 11 or above indicated the probable presence of a mood disorder, and a score of 8–10 merely implied the presence of the relevant condition. Aydemir et al. [27] assessed the validity of the Turkish version of the HAD.

#### 2.2.5. Borg Fatigue Scale

The Borg CR10 Fatigue Scale was used to subjectively assess the exercise-related fatigue levels. This scale is a categorical measurement tool that allows participants to rate their perceived fatigue or exertion level on a scale ranging from 0 (not at all tired) to 10 (extremely tired, maximum fatigue). It is particularly useful for assessing respiratory difficulty, muscle fatigue, and exercise intensity. The CR10 scale is derived from the original Borg 6–20 scale and is widely preferred in clinical applications because of its brevity and ease of understanding by participants. The validity and reliability of the scale have been tested in various clinical and healthy populations and have been reported to show a high level of correlation with objective physiological parameters, such as heart rate, oxygen consumption, ventilation, and lactate levels [28]. In this study, measurements were taken both before and after exercise to assess changes in the participants’ perceptions of physical fatigue.

#### 2.2.6. Tanita Body-Fat Analysis (Bioelectrical Impedance Analysis (BIA))

Bioelectrical impedance analysis (BIA) was performed using a Tanita-305 body fat analyzer (Tanita Corp., Tokyo, Japan), which provides the output of measured impedance and calculated body fat. The participants stood on the metal base plates of the machine while wearing swimsuits. All measurements were taken after a standing period of at least 10 min to minimize potential errors caused by sudden shifts in fluid distribution. Regardless of the participants’ exercise habits, the body composition of all participants was predicted using standard prediction equations rather than those specifically designed for athletes. However, as expected for a general population sample, very few subjects qualified for expert classification. The details of the prediction equations were provided by the manufacturer of the device. The prediction equation for men was derived based on body density (BD) as follows:

BD = 1.100696 − 0.107903 × Wt × Z/Ht2 + 0.00017 × Z

where Wt is weight (kg), Ht is height (m), Z is impedance (Ω), and percentage fat is calculated from body density as

FFM (kg) = 13.96674 + 0.348613 × Ht2/Z + 0.168998 × Wt

For women, the prediction equation estimates fat-free mass (FFM) as

FFM (kg) = 13.96674 + 0.348613 × Ht2/Z + 0.168998 × Wt

The percentage fat was calculated as follows:

(Wt − FFM)/Wt × 100

#### 2.2.7. Chronic Respiratory System Issues Questionnaire (CRSIQ)

The CRSIQ is a questionnaire that measures both physical and emotional health perceptions of the patient. The CRSIQ evaluates four aspects: dyspnea, fatigue, emotional function, and disease mastery. Each area contains 4–7 items, and each item is rated on a 7-point Likert scale; the item scores in each area are summed. The original CRQ, an interviewer-administered instrument, contains 20 items across four domains: dyspnea (five items), fatigue (four items), emotional functioning (seven items), and mastery (four items). When completing this instrument, patients rate their experiences on a 7-point scale ranging from 1 (maximum impairment) to 7 (no impairment). Lower scores indicate problems within the respiratory system [28].

### 2.3. Statistical Data Analysis

Categorical data were summarized as frequencies (percentages). The normality of the distribution of quantitative data was assessed using the Shapiro-Wilk test. Quantitative data were summarized as median (minimum-maximum) and mean ± standard deviation (SD). In the statistical analyses, categorical variables were compared using Pearson’s chi-square, Yates-corrected chi-square, and Fisher’s exact chi-square tests. For quantitative variables, comparisons between two independent groups were made using the Mann-Whitney U test based on the normality of the distribution. When significant differences were detected, Cohen’s d effect size was calculated to determine the effect magnitude. Effect size interpretation was based on Cohen’s d values, where small, medium, and large effects were considered to be between 0.20 and 0.50, 0.50 and 0.80, and above 0.80, respectively [29].

Multivariate analysis was performed using Permutational Multivariate Analysis of Variance (PERMANOVA) to evaluate the overall differences between groups across psychological variables and LCADL categorical variables. Three PERMANOVA models were compared: (1) psychological variables only, (2) LCADL categorical variables only, and (3) a combined model. The analysis used appropriate distance metrics (Bray-Curtis, Hamming, and Gower) for different data types and was performed with 999 permutations. Principal Component Analysis (PCA) was applied to visualize and validate the results.

Statistical significance was set at *p* < 0.05. Univariate analyses were performed using IBM SPSS Statistics 26.0 for Windows (New York, NY, USA), and multivariate analyses and visualizations were conducted using Python 3.10.

## 3. Results

According to the findings in Table 1, in terms of symptoms, 83.67% of the COPD group had cough, and 87.76% had sputum and wheezing, whereas these symptoms were not observed at all in the CG. Smoking was observed in 32.65% of the patient group, while this rate was 7.84% in the CG. According to the MRC dyspnea scale, 98.04% of the CG had no dyspnea, 53.06% of the patient group had severe dyspnea, and 28.57% had moderate dyspnea.

As shown in Table 2, a substantial proportion of patients with COPD reported limitations in their daily living activities. Specifically, 48.98% experienced difficulty in personal care, 83.67% in physical activity, and 36.73% in leisure-related tasks. In contrast, the participants in the CG reported virtually no limitations in these domains. However, no statistically significant difference was observed between the groups regarding difficulty with household chores (*p* = 0.238), suggesting that this specific domain may be less sensitive to the functional impairments associated with COPD than the other domains.

The findings in Table 3 revealed significant differences between the COPD and CG in terms of body composition, questionnaire scores, exercise parameters, and pulmonary function tests. In the body composition assessment, the waist-hip ratio [0.94 (0.82–1)] of COPD was found to be significantly higher than that of CG [0.87 (0.7–0.98)] (*p* < 0.001). Fat ratio [21 (10.1–36.1)] and fat weight [15 kg (3.5–44)] of the COPD group were lower than that of HG [35 (15.4–42.7) and 24.26 kg (10.58–41.93)], respectively; fluid ratio [56.4 (46.4–66.4)], muscle weight [ 53.8 kg (30.2–74.1)] and fluid weight [ 40.4 kg (29.3–66.4)] values were significantly higher than CG [47.1 (40.6–61.2), 42 kg (34.1–55.2) and 33 kg (28–42.13)], respectively (*p* < 0.001).

Analysis of survey data revealed that participants in the COPD group exhibited significantly worse psychosocial outcomes compared to the CG. Specifically, patients with COPD had markedly lower scores on the CRSIQ [median 4 (range: 0–9)] compared to the CG [38 (15–45)], indicating a greater symptom burden. Additionally, anxiety [8 (2–15)] and depression [11 (0–21)] scores, as measured by the Hospital Anxiety and Depression Scale (HADS), were significantly elevated in the COPD group relative to the CG [anxiety: 5 (2–10); depression: 5 (3–11)] (*p* < 0.001 for CRSIQ and depression; *p* = 0.006 for anxiety). These findings underscore the profound impact of COPD on patients’ psychological well-being and quality of life.

In the evaluation of exercise parameters, no significant difference was found between the groups in terms of resting heart rate [COPD: 83 (56–107), CG: 76 (59–116), *p* = 0.057] and end heart rate [COPD: 113 (67–130), CG: 103 (37–130), *p* = 0.166], while SpO2 values were significantly lower in the COPD group both at rest [COPD: 94 (64–97), CG: 97 (94–100)] and at the end of exercise [COPD: 91 (70–96), CG: 95 (84–98)] (*p* < 0.001). Similarly, Borg fatigue scores were significantly higher in the patient group both at rest [0 (0–4)] and at the end [4 (0–7)] than in the CG [0 (0–3) and 0 (0–3) respectively] (*p* < 0.001).

Pulmonary function test (PFT) results demonstrated a significant decline in respiratory capacity in patients with COPD compared to the CG. Median values for the COPD group were as follows: FVC-L [1.83 (1.09–2.86)], FVC% predicted [47% (35–98)], FEV1 [1.1 (0.65–2.7)], FEV1% predicted [40% (24–70)], FEV1/FVC ratio [65.1 (50.9–75.2)], PEF-L [1.97 (1.16–4.01)], and PEF% predicted [32% (14–49)]. All of these values were significantly lower than those observed in the CG: FVC-L [2.29 (1.63–3.02)], FVC% [75% (60–102)], FEV1 [2.16 (1.2–2.57)], FEV1% [92% (54–115)], FEV1/FVC ratio [92.1 (73.6–998.8)], PEF-L [4.08 (2.25–7.32)], and PEF% [72% (40–116)] (*p* < 0.001 for all comparisons). These results confirm a marked deterioration in pulmonary function in individuals with COPD.

Comparative PERMANOVA systematically compared three different model approaches to evaluate the differences between the COPD and control groups (Table 4). In the psychological variables only model (anxiety, depression, CRSIQ), a strong group separation was observed with an F-statistic of 98.12 and an explained variance ratio (R^2^) of 0.50 (*p* < 0.001). Remarkably, the model containing only categorical LCADL variables also demonstrated similar discriminatory power (F = 100.55, R^2^ = 0.51, *p* < 0.001), indicating that limitations in daily living activities independently play a critical role in group separation. The combined model (psychological + LCADL) exhibited the highest statistical power (F = 116.35, R^2^ = 0.54, *p* < 0.001), revealing that adding LCADL categories provided a significant 8.5% contribution to the psychological variable model.

**Figure 1:**
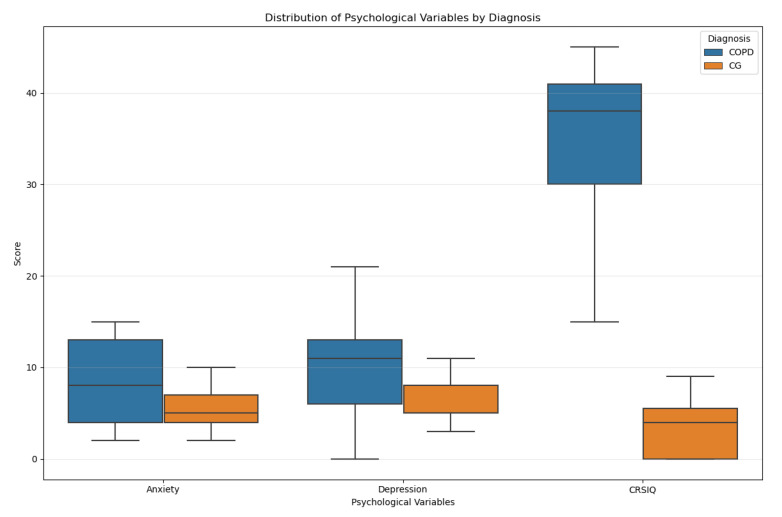
Box plots of psychological variables by diagnosis.

The strong discriminatory performance of the psychological variables model (R^2^ = 0.50) and its underlying group differences can be examined in more detail through the distribution characteristics of the variables (Figure 1). Box plots showing the distribution of psychological variables (anxiety, depression, CRSIQ scores) between COPD patients and CG are given in Figure 1.

**Figure 2:**
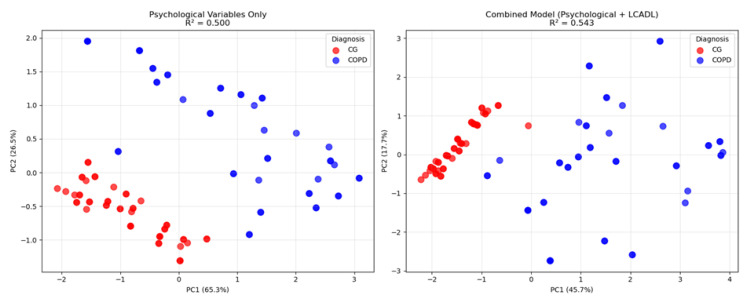
Principal Component Analysis comparison showing group separation between patients with COPD and the CG. (**Left panel**): psychological variables only (anxiety, depression, CRSIQ) with R^2^ = 0.50. (**Right panel**): combined model (psychological + LCADL categories) with R^2^ = 0.54. Blue circles = CGs; Red squares = patients with COPD.

To further validate these statistical findings and visualize the enhanced discriminatory power achieved by combining psychological and LCADL variables, Principal Component Analysis was employed to demonstrate the multivariate separation patterns between groups (Figure 2).

**Figure 3:**
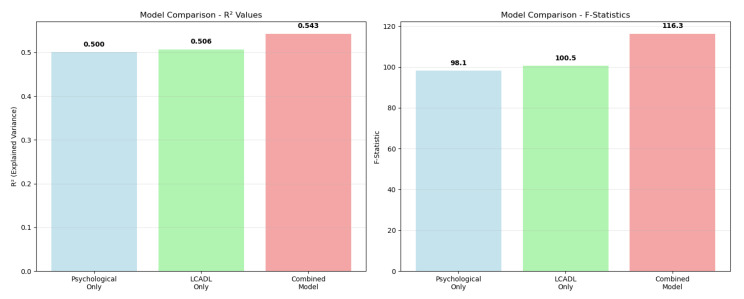
Comparative analysis of PERMANOVA model performance. (**Left panel**): R^2^ values showing the variance explained by each model. (**Right panel**): F-statistics demonstrating the statistical power of each approach. The values are displayed above each bar.

To provide a comprehensive overview of the relative performance and statistical power of all three PERMANOVA models, a comparative analysis of their key metrics was performed (Figure 3).

Comparative visualization of PERMANOVA model performance systematically revealed the contributions of different variable combinations to group separation (Figure 3). In the R^2^ value comparison, the psychological variable model (R^2^ = 0.50) and LCADL category model (R^2^ = 0.51) demonstrated similar discriminatory power. However, the combined model achieved the highest performance (R^2^ = 0.54), demonstrating its synergistic superiority. The F-statistic comparison also supported the same pattern, showing that the combined model had the strongest statistical effect (F = 116.35). This visual analysis concretely demonstrates that a holistic approach to COPD patient assessment provides methodological superiority.

## 4. Discussion

The main findings of this study were that patients with COPD have higher levels of depression and anxiety than healthy controls, difficulty performing daily living activities, increased fatigue levels, and decreased muscle strength. The weight of the COPD group was lower than that of the CG, but their levels of anxiety and depression were higher. Patients with COPD also show a lower maximal aerobic capacity. COPD is more common among smokers, and these patients frequently experience wheezing, sputum production, and cough. This study represents a significant methodological advancement in COPD research by implementing a comprehensive multivariate analytical framework that addresses the complex and interconnected nature of the psychological and functional manifestations in patients with COPD. Our systematic application of Permutational Multivariate Analysis of Variance (PERMANOVA) revealed critical insights that would not have been apparent through traditional univariate approaches. The comparative analysis of three distinct models—psychological variables alone (R^2^ = 0.50), LCADL categorical variables alone (R^2^ = 0.51), and the combined model (R^2^ = 0.54)—demonstrated that both psychological and functional domains independently contribute substantial discriminatory power, while their integration achieves optimal classification accuracy with an additional 8.5% variance. This finding has profound clinical implications, as it provides empirical evidence for the necessity of holistic COPD assessment protocols that simultaneously evaluate psychological well-being and functional capacity, rather than treating these domains in isolation.

In the last two decades, the understanding of the importance of nutrition in COPD has led to increased interest in the management of malnutrition in patients with COPD. Although BMI is the most commonly used tool to assess nutritional status in COPD, changes in weight and BMI classification do not account for changes in body composition, including the distribution of fat and lean mass [30]. Dual-Energy X-ray Absorptiometry (DEXA) is a simple, non-invasive method considered a reference technique for evaluating body composition in patients with COPD (Engelen et al., 1998) [10]. However, the current disadvantages of this technique are its cost and accessibility [31]. Performing DEXA on subjects with low autonomy is challenging. Compared to DEXA, the traditional Bioelectrical Impedance Analysis (BIA) system has satisfactory clinical accuracy in predicting body composition [32]. The BIA system, which is based on the varying resistance to electrical current between body tissues, has been used in various studies to assess body composition in patients with COPD [33]. Studies conducted on patients with COPD have reported body composition values, such as fat percentage, fluid percentage, muscle mass, and waist-to-hip ratio. For example, one study reported values of 16%, 54.5%, 56%, and 0.92, respectively, while another study reported values of 17.9%, 56.4%, 49.2%, and 0.93 [32,34]. The differences in body composition observed between patients with COPD and healthy individuals have clinical significance beyond statistical significance. The low fat percentage observed in COPD may serve as an early indicator of pulmonary cachexia, which can develop due to systemic inflammation and energy imbalance [35]. High muscle mass may not reflect an increase in functional strength but rather changes associated with interstitial fluid accumulation, intramuscular fat, or inactivity. Additionally, an increase in the fluid percentage indicates a risk of fluid retention, which may negatively affect respiratory function [36]. Therefore, detailed body composition analysis using BIA enables a comprehensive evaluation of the physical and metabolic status of patients with COPD and contributes to the identification of individuals at risk of sarcopenia or cachexia in the early stages.

Body composition profiles obtained through BIA in patients with COPD have become an important tool for assessing the current physiological status and developing personalized treatment plans. For example, in individuals with low body fat and weight loss, strategies to increase nutritional support and energy intake become a priority, whereas in patients with reduced muscle mass [37], resistance-based exercise programs may be recommended to reduce the risk of sarcopenia [15,38]. However, interventions such as fluid balance, cardiac assessment, and edema management may be required in patients with elevated body fluid levels. In this context, BIA supports the development of intervention plans based on both physiological and functional goals tailored to the heterogeneous clinical findings of patients with COPD. The literature emphasizes that BIA can be used not only for diagnostic purposes but also for monitoring the treatment process in patients with cancer. Therefore, BIA-based assessment models represent a valuable clinical tool that can be at the center of a multidisciplinary approach to COPD management.

The main methods used to assess dyspnea in patients with COPD are the MRC dyspnea scale and the modified Borg scale [39]. The MRC scale examines the patient passively, whereas the modified Borg scale evaluates the patient during exercise using a pulse oximeter. It has been reported that a modified Borg scale is necessary to validate the MRC scale values [40]. In this study, the modified Borg scale scores significantly differed between the COPD and CG. As a result, the fatigue assessment was higher in favor of the COPD group.

Skeletal muscle loss is a strong predictor of mortality in patients with COPD, independent of lung function [40]. In addition to clinical signs and symptoms, pulmonary function test parameters, which are considered objective findings, are important for the diagnosis of COPD [41]. Clinically, rapid deterioration of lean body mass has been described after an acute exacerbation of COPD, especially in patients with more severe disease (FEV1 < 50%) [42]. In our study, the respiratory functional capacity of patients with COPD was significantly lower than that of the CG.

Reduced respiratory functional capacity, increased fatigue levels, and the inability to perform daily activities over time have led to higher levels of anxiety and depression in patients compared to healthy volunteers [2,43]. At the same time, sedentary behavior is highly prevalent in COPD patients, and this behavior is further elevated in the presence of accompanying depression [44]. In our study, anxiety and depression levels were higher in the COPD group. Our results are consistent with those of other studies in the literature.

Patients with COPD have significantly lower daily physical activity levels than healthy controls; they spend significantly less time walking, walk at a lower intensity than their healthy peers, and most do not meet the current recommendations for physical activity levels [5]. In a study, LCADL results with a 28% cutoff were used to distinguish the functional status of patients with COPD [45]. Patients with an LCADL total > 28% were compared to those with an LCADL total ≤ 28% and were associated with worse lung function, dyspnea, health-related quality of life, and overall health status [45]. In our study, the LCADL results were ≤28%. Our findings are consistent with those of the literature.

COPD is a respiratory disease characterized by airway obstruction. Dyspnea, fatigue, and a decline in respiratory function reduce the quality of life in patients with COPD. Studies in the literature support this finding [46].

This study had several limitations. First, the cross-sectional design precludes causal inference between changes in body composition and psychological outcomes. Second, the study included only male participants, limiting the generalizability of the findings to female patients with COPD. Third, physical activity levels and nutritional intake, which may influence body composition and mood, were not directly assessed in this study. Future longitudinal studies incorporating both sexes and a broader range of biopsychosocial variables are warranted.

## 5. Conclusions

This study highlights the complex interplay between altered body composition, diminished physical function, and psychological distress in men with chronic obstructive pulmonary disease. Compared to healthy controls, patients with COPD demonstrated significantly reduced fat mass, increased muscle and fluid content, greater fatigue, impaired daily living activities, and elevated anxiety and depression symptoms. These findings suggest that a comprehensive body composition assessment using bioelectrical impedance analysis (BIA) offers clinically valuable insights beyond traditional pulmonary metrics. Integrating BIA with standardized psychological and functional evaluations may enhance the early identification of high-risk individuals and inform personalized interventions aimed at improving both physiological and psychosocial outcomes in COPD. Future longitudinal and interventional studies, including diverse populations, are needed to further elucidate the causal pathways and optimize multidisciplinary management strategies for this complex condition.

## Figures and Tables

**Table 1:** Distribution of demographic, clinical, and functional characteristics of the CG and COPD groups.

Variables		CG	COPD	*p*-Value
		n (%)	n (%)	
Gender	Male	51 (100)	49 (100)	<0.001 **
Cough	There is	0 (0)	41 (83.67)	<0.001 **
	None	51 (100)	8 (16.33)	
Sputum	There is	0 (0.00)	43 (87.76)	<0.001 **
	None	51 (100.00)	6 (12.24)	
Grunt	There is	0 (0.00)	43 (87.76)	<0.001 **
	None	51 (100.00)	6 (12.24)	
Cigarette	There is	4 (7.84)	16 (32.65)	<0.001 *
	None	39 (76.47)	0 (0.00)	
	He left	8 (15.69)	33 (67.35)	
MRC	Dyspnea: None	50 (98.04)	0 (0.00)	<0.001 *
	Mild dyspnea	0 (0.00)	4 (8.16)	
	Moderate Dyspnea	1 (1.96)	14 (28.57)	
	Severe Dyspnea	0 (0.00)	26 (53.06)	
	Very Severe	0 (0.00)	5 (10.20)	

* Denotes Pearson chi-square test; ** Denotes Yates’s correction chi-square test.

**Table 2:** CG and COPD group LCADL.

Variables		CG	COPD	*p*-Value
		n (%)	n (%)	
Personal Care	Does not attract	51 (100)	25 (51.02)	<0.001 **
	It’s pulling.	0 (0)	24 (48.98)	
Homework	Does not attract	51 (100)	47 (95.92)	0.238 ***
	It’s pulling.	0 (0)	2 (4.08)	
Physically	Does not attract	50 (98.04)	8 (16.33)	<0.001 **
	It’s pulling.	1 (1.96)	41 (83.67)	
Spare time	Does not attract	51 (100)	27 (63.27)	<0.001 **

** Denotes Yates’s correction chi-square test; *** Denotes Fisher’s exact chi-square.

**Table 3:** Comparison of body analysis, questionnaire scores, fatigue, and respiratory function test findings of the HG and COPD groups.

Variables	Diagnosis		*p*-Value *	ES	ES Level
	CG	COPD			
Size	160 (145–170)	170 (150–181)	<0.001	0.395	Small
Weight	68.4 (56.8–98.2)	71.7 (35.5–122)	0.245	–	–
BMI (kg/m^2^)	25 (20.5–38.4)	24.6 (14.7–37.8)	0.661	–	–
Age	60 (50–74)	66 (55–79)	<0.001	0.448	Small
BIA Analysis					
Waist-Hip Ratio	0.87 (0.7–0.98)	0.94 (0.82–1)	<0.001	0.591	Medium
Fat Rate	35 (15.4–42.7)	21 (10.1–36.1)	<0.001	0.671	Medium
Liquid Ratio	47.1 (40.6–61.2)	56.4 (46.4–66.4)	<0.001	0.599	Medium
Muscle Weight	42 (34.1–55.2)	53.8 (30.2–74.1)	<0.001	0.440	Small
Fat Weight	24.26 (10.58–41.93)	15 (3.5–44)	<0.001	0.547	Medium
Liquid Weight	33 (28–42.13)	40.4 (29.3–66.4)	<0.001	0.474	Small
Surveys					
CRSIQ	4 (0–9)	38 (15–45)	<0.001	0.864	Large
Anxiety	5 (2–10)	8 (2–15)	0.006	0.276	Small
Depression	5 (3–11)	11 (0–21)	<0.001	0.454	Small
Fatigue BORG Score					
Pre-PULSE	76 (59–116)	83 (56–107)	0.057	–	–
Pre-SpO2	97 (94–100)	94 (64–97)	<0.001	0.630	Medium
Pre-BORG	103 (37–130)	113 (67–130)	0.166	–	–
Post-PULSE	95 (84–98)	91 (70–96)	<0.001	0.421	Small
Post-SpO2	0 (0–3)	0 (0–4)	<0.001	0.405	Small
Post-BORG	0 (0–3)	4 (0–7)	<0.001	0.784	Medium
Respiratory Function Test					
FVC (L)	2.29 (1.63–3.02)	1.83 (1.09–2.86)	<0.001	0.579	Medium
FVC (%)	75 (60–102)	47 (35–98)	<0.001	0.715	Medium
FEV_1_ (L)	2.16 (1.2–2.57)	1.1 (0.65–2.7)	<0.001	0.768	Medium
FEV_1_ (%)	92 (54–115)	40 (24–70)	<0.001	0.841	Large
FEV_1_/FVC	92.1 (73.6–998.8)	65.1 (50.9–75.2)	<0.001	0.857	Large
PEF (L)	4.08 (2.25–7.32)	1.97 (1.16–4.01)	<0.001	0.748	Medium
PEF (%)	72 (40–116)	32 (14–49)	<0.001	0.836	Large

ES: Effect Size; CRSIQ: Chronic Respiratory System Issues. Variables are presented as median (minimum-maximum) based on the normality of the distribution. *: Mann Whitney you test.

**Table 4:** Comparative PERMANOVA analysis results.

Model	F-Statistic	Degrees of Freedom	R^2^	*p*-Value	Distance Metric
Psychological Only	98.12	1.98	0.50	<0.001	Bray-Curtis
LCADL Only	100.55	1.98	0.51	<0.001	Hamming
Combined Model	116.35	1.98	0.54	<0.001	Gower

LCADL: London Chest Activity of Daily Living; R^2^: Explained variance ratio.

## Data Availability

The original contributions presented in this study are included in the article. Further inquiries can be directed to the corresponding author.

## Abbreviations

GOLD	Global Initiative for Chronic Obstructive Lung Disease
COPD	Chronic Obstructive Pulmonary Disease
PFT	Pulmonary Function Tests
PEF	Peak Expiratory Flow
MRC	Medical Research Council
MVV	Maximum Voluntary Ventilation
FEV_1_	Forced Expiratory volume in minute
